# Genetic variation in *MKL2* and decreased downstream PCTAIRE1 expression in extreme, fatal primary human microcephaly

**DOI:** 10.1111/cge.12197

**Published:** 2013-06-18

**Authors:** EI Ramos, GA Bien-Willner, J Li, AEO Hughes, J Giacalone, S Chasnoff, S Kulkarni, M Parmacek, FS Cole, TE Druley

**Affiliations:** a Center for Genome Sciences and Systems Biology, Washington University School of MedicineSt. Louis, MO, USA; b Department of Pediatrics; c Department of Pathology and Immunology, Washington University School of MedicineSt. Louis, MO, USA; d Penn Cardiovascular Institute, University of Pennsylvania School of MedicinePhiladelphia, PA, USA

**Keywords:** CArG-box binding factor, exome, microcephaly, MKL2, SRF

## Abstract

The genetic mechanisms driving normal brain development remain largely unknown. We performed genomic and immunohistochemical characterization of a novel, fatal human phenotype including extreme microcephaly with cerebral growth arrest at 14–18 weeks gestation in three full sisters born to healthy, non-consanguineous parents. Analysis of index cases and parents included familial exome sequencing, karyotyping, and genome-wide single nucleotide polymorphism (SNP) array. From proband, control and unrelated microcephalic fetal cortical tissue, we compared gene expression of RNA and targeted immunohistochemistry. Each daughter was homozygous for a rare, non-synonymous, deleterious variant in the *MKL2* gene and heterozygous for a private 185 kb deletion on the paternal allele, upstream and in cis with his *MKL2* variant allele, eliminating 24 CArG transcription factor binding sites and MIR4718. *MKL1* was underexpressed in probands. Dysfunction of *MKL2* and its transcriptional coactivation partner, serum response factor (SRF), was supported by a decrease in gene and protein expression of PCTAIRE1, a downstream target of MKL2:SRF heterodimer transcriptional activation, previously shown to result in severe microcephaly in murine models. While disruption of the MKL2:SRF axis has been associated with severe microcephaly and disordered brain development in multiple model systems, the role of this transcription factor complex has not been previously demonstrated in human brain development.

A healthy Caucasian couple with no known common ancestor delivered three live born daughters, from independent conceptions, all with a cadre of severe developmental anomalies, the most striking of which was severe microcephaly 1:20th the size of a typical neonatal brain. The microcephaly was accompanied by intrauterine growth retardation, multiple cardiac anomalies, cleft lip and palate as well as a variety of other developmental anomalies. In all three cases, the children expired during the perinatal period following the withdrawal of cardiorespiratory support. The constellation of findings did not fit any known phenotype or syndrome. Karyotype analysis and high-density, genome-wide single nucleotide polymorphism (SNP) array failed to identify a genetic anomaly that could account for this severe and unusual phenotype. Exome sequencing has previously been successfully employed for the identification of genes underlying rare phenotypes 1. We performed exome sequencing of both parents and all three probands. We identified a candidate gene (*MKL2*, NM_014048) that was homozygous for a rare, non-synonymous point mutation in all three children. In model systems, *MKL2* has previously been associated with brain and cardiac abnormalities due to disruption of the MKL2:SRF transcription factor axis 2–6.

Transcriptional coactivators play critical roles in transducing signals required for embryonic development. The myocardin family of transcriptional coactivators [Myocardin, Myocardin-like Protein 1/Megakaryoblastic Leukemia 1 (*MKL1*, NM_020831) and Myocardin-like Protein 2/Megakaryoblastic Leukemia 2 (*MKL2*)] bind to, and synergize with, the conserved transcription factor, serum response factor (*SRF*, NM_003131) in a variety of tissues. *MKL2* encodes a 1080 amino acid protein that is enriched in the forebrain, particularly the hippocampus and cerebral cortex 2,3. MKL2 and MKL1 share 42% sequence identity with conserved N-terminal actin-binding domains, along with the basic, glutamine-rich, leucine zipper and SAP domains 7. SRF is a MADS box (homology domain in *MCM1*, *Agamous*, *Deficiens*, and *SRF*) transcription factor that binds DNA at the CArG box, containing the consensus sequence CC(A/T)_6_GG, found in the promoters of immediate early genes and a subset of cytoskeletal/myofibrillar genes 8. Heterodimerization between the *MKL*s and *SRF* is mediated through the basic and glutamine-rich domains and the MADS box, respectively. MKL1/2 bind monomeric G-actin in the cytoplasm. Stimulation of Rho-GTPases induces filamentous F-actin polymerization in the cytoplasm, causing dissociation of G-actin from MKL1/2 and relocation of these proteins into the nucleus where they bind to SRF and, through a TGF-β activated signaling pathway, induce gene transcription leading to neurite outgrowth and neuronal morphology 4,9,10.

Model animal systems have demonstrated severe consequences in brain development associated with disruption in *SRF*, *MKL1* and/or *MKL2* expression. Homozygous *Srf*-null murine embryos demonstrated embryonic lethality at about E6.5 due to lack of mesoderm formation with misfolded ectodermal cell layers 5. Conversely, injection of dominant-negative *SRF* constructs in *Xenopus* embryos resulted in a shortened body axis and microcephaly due to loss of ectodermal cell fates 6. In the human brain, SRF expression is restricted predominantly to neurons according to The BrainMap Project (www.brainmap.org), which is consistent with the lack of dendritic complexity observed in neurons from rats that express a dominant-negative SRF mutant 3 and mice with conditionally deleted *Srf* expression that show impaired neurite outgrowth, neuronal migration, axon guidance, and synaptic formation 2,11. Similar decreases in dendritic length and the number of dendritic processes were observed after inhibition of *Mkl1* 11,12 or *Mkl2* 3,13. While *Mkl1*-null mice have no neuronal phenotypes and exhibit only defects in mammary myoepithelium 14, *Mkl2*-null mice demonstrated post-gastrulation embryonic lethality secondary to a constellation of developmental cardiac defects 15. Conditional brain-specific knockout of *Mkl2* in a *Mkl1*^−/−^ murine background phenocopied the neurological abnormalities seen in *Srf*-null mice and led to lethality between postnatal days 16–21 4. Despite these results, no developmental anomalies secondary to perturbations in this pathway have been described in humans.

## Methods

### Genome wide SNP arrays

High-density genome-wide SNP array genotyping was performed by the Washington University Clinical and Molecular Cytogenetics Laboratory from all three probands and both parents using the Affymetrix 6.0 platform with two million tagged SNPs. Raw data were analyzed using the publicly available Affymetrix, Santa Clara, CA; Genotyping Console™ (http://www.affymetrix.com/browse/level_seven_software_products_only.jsp).

### Exome sequencing and analysis

Exome sequence and genome-wide array data reported in this paper can be found at dbGaP accession number phs000553.v1.p1. Exome sequencing of all three probands and both parents was performed using the Agilent SureSelect 38 Mb All Exon Kit according to the manufacturer’s protocol. Sequencing was performed on the Illumina GAIIx platform. Two lanes of 76 bp paired-end sequencing were obtained per person resulting in a range of 10,397,265,576–13,030,190,120 total bases per individual. Raw sequence data were aligned against the human genome (hg18/NCBI build 36) followed by variant calling using algorithms and settings from a previously published bioinformatic pipeline 16.

An average of 144,887,840 raw reads was obtained from each individual with 81% mapping back to reference sequence after the removal of duplicate reads. Sequencing was highly uniform among all samples, with an average of only 1.16% of targets not captured and 96% of all targets surpassing fivefold coverage. By matching called genotypes from exome sequencing with an average of 2937 variant positions per person from microarray results, we found an average sensitivity of 97% (range 96.3–97.3%). By matching called sequencing genotypes against an average of 4337 wild-type positions as determined by microarray, we found an average specificity of 99.8% (range 99.7–99.8%).

### Sanger sequencing of *MKL2* and *SRF* in microcephaly patients

To validate exome sequencing results and survey additional cases, Sanger sequencing of *MKL2* and *SRF* was performed. Polymerase chain reaction (PCR) amplification and sequencing primers were designed using Primer3 (http://frodo.wi.mit.edu/) with previously published settings 17. Amplicons were designed to span all untranslated and coding bases. Primer sequences are listed in Table S3, Supporting Information. Individual PCR products were purified by Qiagen, Valencia, CA; MinElute spin columns and resuspended in TE buffer before sequencing. Results were analyzed using Phred/Phrap/Consed 18.

### Selection of additional microcephaly cases

To determine whether *MKL2* or *SRF* variants occurred in other unrelated cases of microcephaly of varying severity, we performed Sanger sequencing on DNA from 51 unrelated cases with varying degrees of microcephaly from four geographically separate research centers. A search at Washington University from 1991 to 2009 provided cortical brain tissue from 15 fetal and newborn cases with microcephaly (brain weight smaller than clinically-accepted reference ranges for stated age 19) but no other brain abnormalities or global growth retardation. An additional three age-matched cases without history of any brain abnormalities were identified as controls (born between 23 and 26 weeks estimated gestational age and surviving for 2 days to 4 weeks). In addition, thirty-one anonymized DNA samples were obtained from a repository of microcephaly cases at the Institute of Human Genetics, Freiburg, Germany (courtesy of Dr. Deborah Morris-Rosendahl), Four anonymized DNA samples from the University of Washington, Seattle, Washington (courtesy of Dr. William Dobyns), and one DNA sample from a previously published case from Turkey with severe microhydrancephaly 20 whose phenotype was mapped to a locus adjacent to the *MKL2* gene locus (courtesy of Dr. Aslihan Tolun, Bogazici University, Istanbul, Turkey).

### Nucleic acid extraction from the FFPE samples

DNA and RNA extraction was performed from 3 mm needle cores taken from formalin-fixed paraffin-embedded (FFPE) embedded tissue blocks from each case. DNA was retrieved with the PureGene DNA Purification Kit (Gentra, Minneapolis, MN). RNA extraction was performed by Washington University’s Cytogenetics and Molecular Pathology Laboratory.

### Gene expression analysis

We performed real-time quantitative polymerase chain reaction (RT-qPCR) to quantify the relative expression level of *MKL2*, *SRF* and *PCTAIRE1* compared to the housekeeping gene (*ACTB*) in brain from three non-microcephalic controls and three probands. Total RNA was collected from slides of brain sections of formalin-fixed, paraffin-embedded tissue. Given the very thin, underdeveloped proband cortex, we were unable to solely isolate cortical RNA from fixed slides. Total RNA from each sample was reverse transcribed in 20 µl final reaction using random hexamers and the SuperScript III First-Strand Synthesis SuperMix following the manufacturer standard protocol (Invitrogen, Carlsbad, CA). Subsequently, 100 ng of cDNA was amplified in 20 µl of reaction using Brilliant III Ultra-Fast QPCR Master Mix (Agilent Technologies, Santa Clara, CA) according to the manufacturer standard protocol. The PCR protocol was carried out as follows: (1) 95°C for 5 min, (2) 95°C for 15 s, (3) 60°C for 45 s, (4) Read plate, and (5) Repeat steps (2–4) 39 more times.

qPCR probes for each gene were designed using Integrated DNA Technologies (IDT) PrimeTime Mini qPCR Assays. The amplification procedure was carried out in a C1000 Thermal cycler CFX96 Real-Time System (Bio-Rad, Hercules, CA). Samples were run in triplicate using the FAM fluorophore. Statistics were done with a student *t*-test comparing fold expression difference from probands to controls.

### Immunohistochemistry

Hematoxylin and eosin (H&E), DAPI and anti-MKL2 staining were performed in the Parmacek laboratory at the University of Pennsylvania. Anti-MKL2 antibody was from Sigma (#HPA011286; St. Louis, MO). Anti-PCTAIRE1 staining was performed by the Anatomic and Molecular Pathology Core Lab at Washington University using an antibody purchased from Santa Cruz Biotechnology (#sc-166242; Santa Cruz, CA). Staining was performed on one non-microcephalic neonatal control. This individual was born at 29 weeks estimated gestational age and died 14 weeks later.

### Institutional approval

Parents of the probands provided dbGaP compliant, informed consent for themselves and their infant daughters. This study was reviewed and approved by the Washington University Human Research Protection Office (protocol #201102181).

## Results

### Clinical presentation

**Figure 1:**
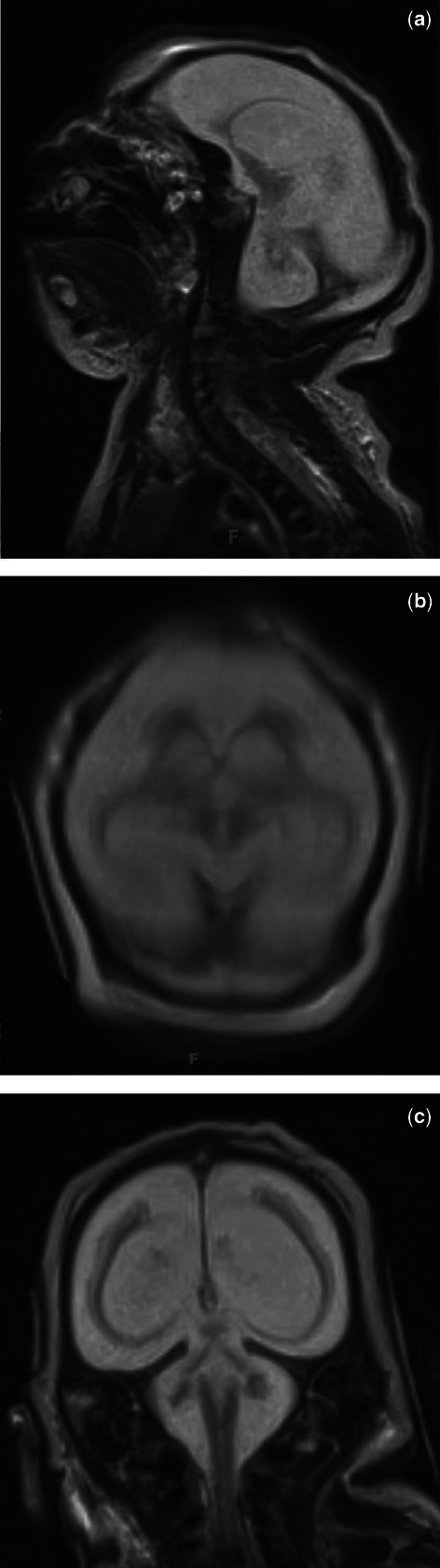
Representative non-contrast T2 brain MRI of one proband. These images are from Proband 2, born at 37 4/7 EGA and taken on day of life two, one day prior to expiration and two days prior to neuroautopsy. These images show sagittal (**a**), axial (**b**) and coronal (**c**) views. In all three probands, MRI demonstrated significant disruption of normal brain architecture including extreme microcephaly, marked cerebellar hypoplasia, and complete lissencephaly with failure of normal opercularization. Although the hypothalamus and optic nerves were present, the pituitary fossa was filled by fat signal, and the brainstem and cerebellum were hypoplastic.

Over a 12-month period, a non-consanguineous, Caucasian couple of European descent (33 year old woman and 35 year old man) without prior pregnancies had a spontaneous singleton pregnancy and a Clomid-induced, dizygous twin pregnancy. Clinical presentation of each infant is summarized in Table 1. All three female infants carried antenatal diagnoses of intrauterine growth restriction and microcephaly and expired within the first four days of life. Multiplanar, multi-weighted, non-contrast magnetic resonance imaging (MRI) of the brain and brainstem of all probands demonstrated significant disruption of normal brain architecture including extreme microcephaly, marked cerebellar hypoplasia, and complete lissencephaly with failure of normal opercularization (Fig. 1). Non-contrast brain MRI analysis of both parents revealed normal brain architecture (not shown).

**Table 1:** Clinical presentation summary

	Proband 1	Proband 2	Proband 3
EGA at birth (weeks, days)	35, 5/7	37, 4/7	37, 4/7
Day of life at expiration	2	3	4
Body length (cm)	38.0 (normal: 43.4 ± 5.9)	45.2 (normal: 45.6 ± 5.1)	45.5 (normal: 45.6 ± 5.1)
Body weight (g)	1400 (normal: 2280 ± 615)	1450 (normal: 2634 ± 534)	1850 (normal: 2634 ± 534)
Head circumference (cm)	24.0 (<3 percentile)	22.6 (<3 percentile)	25.6 (<3 percentile)
Brain weight (g)	14.0 (normal: 278 ± 96)	18.0 (normal: 318 ± 106)	23.5 (normal: 318 ± 106)
Other common findings	• Only Sylvian and interhemispheric sulci		
	• Persistent subpial granular layer		
	• Thin, immature cortical mantle		
	• Gray and white matter gliosis		

EGA, estimated gestational age.

Postmortem examination suggested disruption of brain development at 14–18 weeks gestation. Severe microcephaly (≪3 percentile) with symmetric cerebral hemispheres and sulcation limited to the interhemispheric and Sylvian fissures was observed in all three probands. The cerebral mantle was thin with no macroscopic distinction between white and gray matter and dilated ventricles. No areas of infarction, necrosis, or calcification were noted.

Microscopic and histochemical analyses showed hypocellular cortex with variable cortical lamination and neurofilament staining demonstrating a highly dense axonal plexus that resided at the innermost edge of the moderately cellular subpial granular layer. Internal to this was a second layer with a moderate number of small, primitive-appearing cells with minimal cytoplasm. Under this layer, a third paucicellular layer was visible, and followed by a fourth moderately cellular layer. These cells were well-differentiated and showed primary dendrite formation. Most of these cells stained positive for neuronal nuclear antigen (NeuN). Glial fibrillary acidic protein (GFAP) stain identified numerous reactive astrocytes throughout the cortex. White matter was primitively formed and showed numerous immature oligodendrocytes in various phases of myelination. Nests of primitive, proliferating (Ki-67 positive) cells were primarily located around blood vessels.

According to neuroautopsy reports, the ventricular lining showed normal ciliated columnar epithelium. The caudate/putamen were immature but relatively well formed. The midbrain showed a lack of gray and white matter differentiation with normal formation of the oculomotor nuclear complex, but a paucity of descending tracts. The pons and medulla showed some rudimentary development and a consistent lack of descending tracts. The cerebellum showed poorly developed cerebellar folia with minimal sulcation. There was also a lack of gray and white matter differentiation as well as no Purkinje cell layer or internal granular layer. The spinal cord was difficult to assess due to the absence of white and gray matter differentiation and a lack of descending tracts.

### Karyotype and genome-wide array results

Table 2 lists the copy number variants identified in all three probands. G-banding analysis of metaphase cells extracted from whole blood showed that all probands had female karyotypes and a large deletion on the X chromosome (∼1.63 Mb at Xp22.31), identical to their father. This deletion includes four genes (*HDHD1A*, *PNPLA4*, *STS*, and *VCX*) and two miRNAs (MIR651 and MIR4767). Their father has X-linked icthyosis which is caused by a loss of the *STS* gene 21. All three probands and their father also carried a 185 kb deletion 1.2 Mb upstream of the *MKL2* on chromosome 16p13.12, which is in cis with the paternal *MKL2* variant allele. This private deletion contains 24 CArG boxes of variable regulatory potential and one entire gene, *CPPED1* along with MIR4718 (Fig. S1). There is no reported connection between *CPPED1* and brain development. According to the miR Database (http://mirdb.org), MIR4718 has 156 known targets and two, microcephalin 1 (*MCPH1*, NM_024596) and strawberry notch homolog 1 (*SBNO1*, NM_018183) have known connections to human microcephaly 22–24.

**Table 2:** Copy number variations in probands as determined by karyotype and genome-wide array analysis

	Karyotype	Chromosome	Deletion/ Duplication	Size (kb)	Gene (RefSeq Accession Number)
Proband 1	46, XX	16p13.12	Deletion	185	*CPPED1* (NM_018340)
					*MIR4718* (NR_039869)
		Xp22.31	Deletion	1628	*HDHD1A* (NM_012080)
					*STS* (NM_000351)
					*VCX* (NM_013452)
					*PNPLA4* (NM_004650)
Proband 2	46, XX	16p13.12	Deletion	185	*CPPED1* (NM_018340)
					*MIR4718* (NR_039869)
		22q12.3	Deletion	133	*LARGE* (NM_133642)
		Xp22.31	Deletion	1628	*HDHD1A* (NM_012080)
					*STS* (NM_000351)
					*VCX* (NM_013452)
					*PNPLA4* (NM_004650)
Proband 3	46, XX	4q32.1	Deletion	450	*TDO2* (NM_005651)
					*GUCY1A3* (NM_001130686)
					*GUCY1B3* (NM_000857)
					*CTSO* (NM_001334)
		8p23.2	Duplication	678	*CSMD1* (NM_033225)
		16p13.12	Deletion	185	*CPPED1* (NM_018340)
					*MIR4718* (NR_039869)
		Xp22.31	Deletion	1628	*HDHD1A* (NM_012080)
					*STS* (NM_000351)
					*VCX* (NM_013452)
					*PNPLA4* (NM_004650)

No other copy number variations (CNVs) were shared by the three probands, however, proband 2 had an interstitial 133 kb deletion at chr22q12.3, including the *LARGE* gene, which is associated with structural brain defects 25. To exclude the possibility that all three probands carried the same maternal X chromosome with variants that explain the phenotype, we used single nucleotide variant (SNV) analysis from exome sequencing and SNP array and found that the daughters did not receive the same maternal X chromosome, and there were no maternal X chromosome homozygous variants included in the paternal X chromosome deletion.

### Exome sequencing results

We filtered the exome sequencing data using a bioinformatic pipeline with a published sensitivity and specificity of 96.9% and 99.8%, respectively, when compared to common positions available in Affymetrix 6.0 array data 16. In addition, Sequenom genotyping from all family members was performed at 127 variant positions not represented on the Affymetrix array, including the *MKL2* variant position. Overall, the Pearson correlation between SAMtools and Sequenom validation was *R*^2^ = 0.978 and confirmed *MKL2* heterozygous genotypes in each parent and homozygous genotypes in each proband.

We identified three genes with a homozygous variant (*DPYD*, NM_000110; *TMEM132E*, NM_207313; *MKL2*), and no genes with a compound, non-synonymous genotype. Variants in *TMEM132E* (rs4795954, MAF 13.2%) and in *DPYD* (rs2297595, MAF 6.1%) are common, and neither has been associated with brain development or microcephaly. There were three genes with two variants on the paternal allele (*KALRN*, *SPATA16*, and *TCHH*). Maternal alleles were wild-type for these three genes. Thus, while *KALRN* has been implicated in synaptogenesis 26, the fact that the father has normal cognition and brain morphology caused us to eliminate this variant allele from further analysis. In addition, there were no variants in the *NDE1* locus, which is adjacent to the *MKL2* locus (16p13.11 and 16p13.12, respectively) and has been associated with severe microcephaly with lissencephaly 27–29. Similarly, no variants were detected in *WDR62*, which has also been associated with severe cortical brain malformations 30.

However, both parents were heterozygous for an *MKL2* variant in exon 10 (rs75963814 at chr16:14241627) which encodes a predicted deleterious amino acid substitution from CAC(His)→CAA(Gln) at amino acid 288 and is rare with a reported Caucasian MAF of 0.6% in 1000 Genomes Phase I (http://www.ncbi.nlm.nih.gov/projects/SNP/) and 1.1% in 10,639 exomes on the Exome Variant Server (EVS; http://evs.gs.washington.edu/EVS). This variant falls at the first base of the MKL2 basic domain, which along with the glutamine-rich domain, is required for proper heterodimerization with SRF. However, the *MKL2* missense allele (His288Gln) is present at a population frequency too high to be solely responsible for the rare, extreme phenotype in our probands. The predicted homozygote frequency is 0.02%, or 1:5000. The EVS reports two homozygous His288Gln genotypes among the 10,639 exomes. One of these individuals is an adult female who does not carry any upstream deletion of *MKL2* (S. Kathiresan, personal communication, 2012). We have not identified the other individual.

Assuming independent segregation, three successive conceptions with the same homozygous genotype would be expected to occur with a likelihood of 1:64. To determine if the *MKL2* missense allele was overrepresented in either parent’s germline, we examined the percentage of total reads containing variant versus wild-type genotype. We found that the variant:wild-type allele distribution frequencies in the father’s (37%:63%, respectively) and the mother’s (50%:50%) autosomal exome samples did not support a model of non-independent segregation. To further examine the possibilities that the *MKL2* missense allele frequency differed between father’s sperm and his autosomal exome and that *de novo*, putatively functional variants occurred during spermatogenesis, we sequenced the exome of the father’s sperm. We found the *MKL2* variant present at a frequency of 33%, closely matching the frequency of the variant in his autosomal exome, and found no additional variants in the sperm exome that could account for the probands’ central nervous system phenotype (Table S1).

### Sanger sequencing of other microcephaly cases

To determine whether *MKL2* or *SRF* variants occurred in other unrelated cases of microcephaly of varying severity, we first sequenced *MKL2* exon 10 (*N* = 11) and *SRF* untranslated region (UTR) and coding regions (*N* = 15 including 11 sequenced at *MKL2* and four additional cases) from DNA isolated from FFPE sections of fetal (>20 weeks gestation) and neonatal brain tissue collected at Washington University (Table S2). We found no individuals with exon 10 *MKL2* sequence variants and two individuals with *SRF* variants (individuals 10 and 12). Individual 10 was heterozygous for a novel, noncoding variant with high regulatory potential based on ESPERR score 31. Individual 12 was homozygous for a novel C→T substitution in the 3′ UTR at a highly conserved base (chr16:43,256,479) with moderate predicted regulatory potential.

We also sequenced all UTR and coding regions of *MKL2* and *SRF* in 36 additional cases of severe microcephaly from three geographically distinct centers (see Methods). Although 10 individuals (28%) had at least one synonymous variant in either of these genes (Table S2), three individuals were found to have multiple *MKL2* variants of variable frequencies and predicted regulatory implications. One individual was homozygous for a rare variant (rs140275336) in the 5′ UTR that abolishes a methylated cytosine at a highly conserved base with a high regulatory potential, and a different individual was heterozygous for the same variant. Unfortunately, there was no RNA or tissue available for these individuals to characterize gene expression.

### Expression results

**Figure 2:**
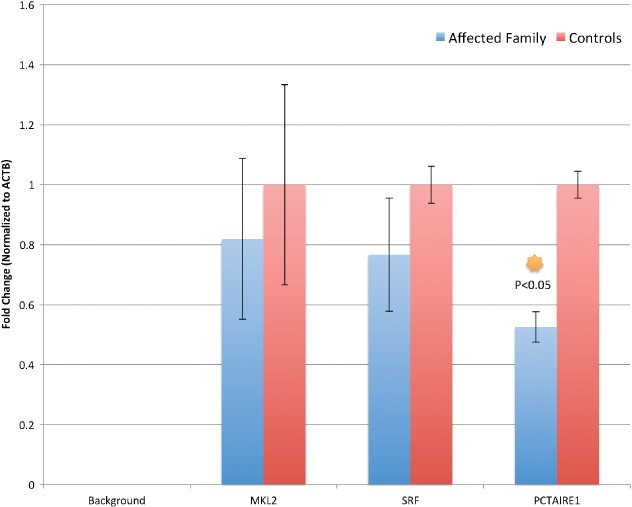
Relative brain cortical gene expression. Controls were three fetal brain tissue specimens without a pathology diagnosis of microcephaly. ‘Affected Family’ is an average of gene expression from all three affected probands.

We performed gene expression analysis of *MKL2*, *SRF* and *PCTAIRE1* via RT-qPCR on three non-microcephalic controls and three probands (Fig. 2). We observed no significant decrease in *MKL2* or *SRF* expression compared to non-microcephalic controls (fold change: 0.82 *vs* 1.0 and 0.77 *vs* 1.0, respectively). However, *PCTAIRE1* showed a significant decrease in expression (fold change: 0.52 *vs* 1.0) and a p-value < 0.05. To survey expression of additional genes associated with the deleted region and/or microcephaly, Affymetrix Quantiplex gene expression was performed (Fig. S2). We again noted no significant difference in *MKL2* gene expression.

### Immunohistochemistry of human control and proband cortex

**Figure 3:**
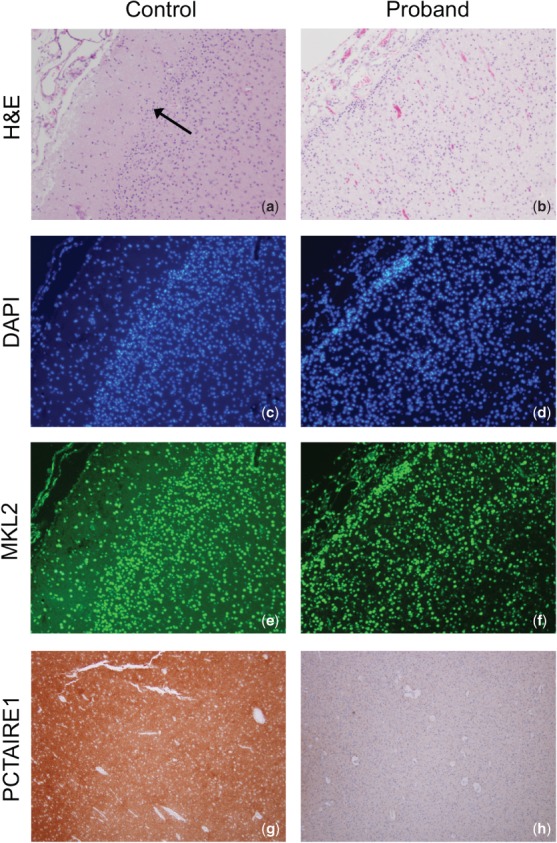
Comparative immunostaining between a non-microcephalic control and proband. Proband specimen is from neuroautopsy after expiration on day of life 4. (**a, b**) H&E staining of cerebrum (×100). In control, note the junction between white and gray matter, which is less evident on the proband image (arrow). (**c, d**) DAPI staining (×100) showing equivalent cell numbers between proband and control. (**e, f**) Nuclear MKL2 immunostaining (×100) is observed in a subpopulation of neurons of control and mutant brains (not all cells in either sample). (**g, h**) Anti-PCTAIRE1 (CDK16) staining of control and proband cortex (×40).

Given that we identified a coding variant within the MKL2 open reading frame and had no evidence for regulatory disruption, there was no *a priori* reason to expect a decrease in MKL2 protein expression. To test this, we performed immunostaining of MKL2 (Fig. 3e,f), SRF (not shown) and PCTAIRE1 (Fig. 3g,h) on proband and control cortex. We identified a subpopulation of neurons that express presumably dysfunctional MKL2 in a pattern similar to human fetal controls with no clear difference in cell numbers, but the cortical rim in probands was much thinner than the cortex in non-microcephalic controls. To investigate putative dysfunction of MKL2:SRF transcriptional activation, control and proband immunostaining with anti-PCTAIRE1 (CDK16, NM_006201) demonstrated a decreased cortical PCTAIRE1 staining in our probands (Fig. 3g,h). No difference in MKL2 staining along with a decrease in PCTAIRE1 protein between probands and controls is consistent with our RT-qPCR results (Fig. 2) as well as similar results in microcephalic mice with conditionally deleted Mkl1/Mkl2 expression 4. Similar cortical staining with anti-phosphorylated CDK5 and anti-TGF-β did not show qualitative differences in protein expression between proband and control cortex (not shown).

## Discussion

We present an extreme human phenotype that includes developmental anomalies of the heart and lungs and lethal microcephaly with apparent brain growth arrest between 14 and 18 weeks. Given the uniqueness of the genotype and severity of the phenotype, there was no unrelated human with an identical phenotype available for genotypic comparison. We suggest that disruption of the *MKL2:SRF* gene transcription pathway, perhaps through diminished MKL2 heterodimerization with SRF due to the point mutation in the basic domain followed by the lack of critical CArG binding domains removed by the private deletion, accounts for extreme microcephaly in the affected infants and support this conclusion with the observation of reduced *PCTAIRE1* gene and protein expression. A lack of proper protein function is also supported by decreased anti-PCTAIRE1 staining, similar to functional studies in animal models resulting in severe microcephaly 4.

In the mouse model, *Mkl2* was expressed during early fetal development as the double knockout was embryonic lethal. Unlike peripheral tissue phenotypes, the brain phenotype was not evident until all four *Mkl1* and *Mkl2* alleles were deleted suggesting brain-specific redundancy in their function. Of note, these double knock-out mice demonstrated a 25% decrease in Srf mRNA, equivalent to the *Srf* mRNA decrease seen in *Srf*^+/−^ mice without brain dysmorphology. These findings suggest that, while *Mkl*1/*Mkl2* regulate *Srf* transcripton, the murine brain phenotype was not due to transcriptional downregulation of *Srf* 4. Instead, these defects were thought to arise due to the inability of the MKL:SRF complex to activate *SRF*-specific genes critical for actin-based remodeling of dendrites and synapses including *Pctaire1*.

Additional data suggest that a lack of MKL:SRF signaling could impair two additional interconnected pathways important for neurodevelopment. Epithelial–mesenchymal transition (EMT) is an important step in embryonic neurodevelopment that is mediated through transforming growth factor-β1 (TGF-β1) activation of Rho-signaling. This activation is required for expression of SRF-dependent cytoskeletal or Smad3-dependent genes via translocation of MKL into the nucleus 9. In Xenopus embryos, loss of SRF function resulted in an extension of mesoderm toward ectoderm by failing to outcompete for Smad2/FAST-1 binding between Activin and Nodal, two TGF-β superfamily members 6. Activin has also been shown to promote SRF- and MKL-dependent dendritic complexity in rat cortical neurons 3. More recently, murine *Mkl*^−/−^ embryonic stem cells were shown to be down-regulated for *TGF-β* expression and signaling, as well as *TGF-β* regulated extracellular matrix genes 32. The MKL:SRF pathway and the *TGF-β* pathway are also known to interact with *Cdk5*, a serine/threonine kinase critical for neuronal development, survival, and regulation of neuronal apoptosis 33. *Cdk*^−/−^ mice exhibited a lack of cortical laminar structures and cerebellar foliation due to defective neurofilament transport and associated with perinatal mortality 34. Reduced expression of Pctaire1, results in a lack of phosphorylated *Cdk5* in mice with severe cortical dysmorphology 4. Lastly, reduced Cdk5 expression in mice with a conditional *TGF-β1*^−/−^ deletion in the trigeminal ganglia and dorsal root ganglion established a direct link between *TGF-β1* and *Cdk5* pathways 35. While we observed a decrease in Pctaire1 protein expression, normal pCDK5 staining suggests an alternative pathway that facilitates CDK5 phosphorylation in human brain development.

**Figure 4:**
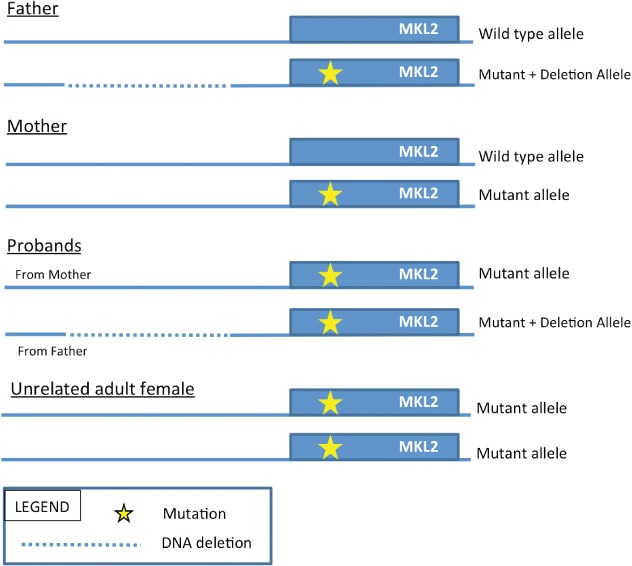
Proposed partial genetic model for variant haploinsufficiency.

Given that the upstream deletion is private to our pedigree, we have not identified an exact genotypic and phenotypic match to our probands. We propose the partial genetic model depicted in Fig. 4. On the basis of the four possible genotypes observed, we hypothesize that the missense *MKL2* allele is hypomorphic, and that the upstream deletion of 24 CArG boxes and/or MIR4718 results in downstream dysregulation of the MKL:SRF heterodimer resulting in a variety of developmental defects. Either one wild-type allele or two hypomorphic alleles could rescue normal brain development, but the combination of hypomorphic alleles and the deletion of regulatory elements results in significant disruption of normal brain development and architecture. While this model is speculative, our observations are consistent with MKL:SRF model systems and have not been previously reported in humans. Our results do not definitively establish causality, and confirmation of the role of MKL:SRF disruption in normal human brain development will require cell and organism based studies of analogous genetic lesions followed by a detailed quantification of MKL2:SRF binding and subsequent gene transcription.

## Supporting Information

The following Supporting information is available for this article:

*Fig. S1*. University of California at Santa Cruz Genome Browser snapshot of the upstream paternal deletion in cis with the paternal variant *MKL2* allele. Twenty-four CArG boxes upstream of *MKL2* are lost in the paternal 185 kb deletion. Many of which overlie regions of high regulatory potential (peaks along the ‘ESPERR Regulatory Potential’ track).

*Fig. S2*. Relative brain cortical gene expression. Relative expression of 27 genes that included those known to be involved in the *SRF:MKL2* pathway, those previously identified in cases of primary microcephaly, and *CPPED1* located in the upstream chromosome 16 deletion and the housekeeping genes *ACTB* and *PPIB* to normalize expression was measured using the Quantigene Plex 2.0 Assay (Affymetrix). Thirteen individuals were surveyed: three normal brain anatomy fetal controls, three affected probands, and seven fetal specimens with pathology-diagnosed microcephaly. For each specimen, total RNA was extracted from an aggregate of six FFPE cerebral cortex cross sections affixed to glass slides according to the manufacturer’s protocol. The average cross-sectional area was 27.6 cm^2^ (range 9.0–49.5). Lysates of each sample were prepared by incubating each sample at 65°C for 6 h with 1 min of full speed vortexing every 60 min. Target-specific probes were designed and provided by Affymetrix. Target hybridization and signal amplification were performed according to manufacturer’s protocol. Signal measurements were made using a Luminex instrument (Austin, TX). Transcripts from each gene in all samples were measured in triplicate, and results expressed as the geometric mean normalized to an aggregate of *ACTB* and *PPIB* expression. Controls were six fetal brain tissue specimens without a pathology diagnosis of microcephaly. ‘Family’ is an average of gene expression from all three affected probands. ‘Other’ is an average of 33 other fetal cases with a pathology diagnosis of microcephaly. *p < 0.05; **p < 0.005. The red * is for comparison of ‘other’ microcephaly cases to normal controls.

Table S1. Based positions analyzed in the paternal sperm exome.

Table S2. Sequencing results summary of *MKL2* and *SRF* from 51 unrelated and geographically distinct primary microcephaly cases. Twelve cases were found to harbor sequence variants. The minor allele frequency, conservation (PhyloP) score and 7-species regulatory potential (ESPERR) score are listed.

Table S3. PCR primers for candidate region amplification and Sanger sequencing.
