# Supplementary material for: Genetic variation in MKL2 and decreased downstream PCTAIRE1 expression in extreme, fatal primary human microcephaly
Source: Clin Genet. 2013 Jun 18;85(5):423–32. doi: 10.1111/cge.12197 (PMC3929543; doi:10.1111/cge.12197)
Supplement: Supplementary file 1 — Fig. S1. University of California at Santa Cruz Genome Browser snapshot of the upstream paternal deletion in cis with the paternal variant MKL2 allele. Twenty-four CArG boxes upstream of MKL2 are lost in the paternal 185 kb deletion. Many of which overlie regions of high regulatory potential (peaks along the ‘ESPERR Regulatory Potential’ track). [file cge0085-0423-sd1.doc]

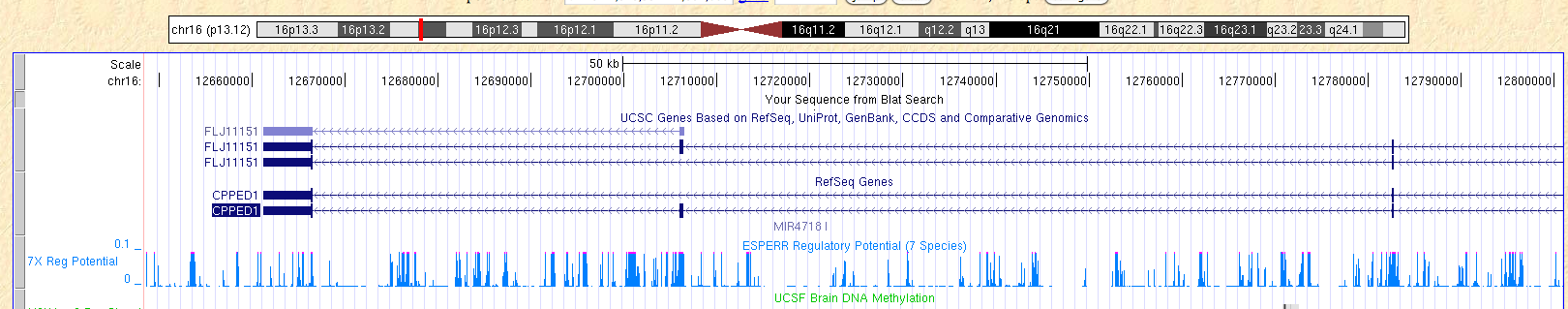


**Supplementary Figure 1. UCSC Genome Browser snapshot of the upstream paternal deletion in cis with the paternal variant *MKL2* allele.** Twenty-four CArG boxes upstream of *MKL2* are lost in the paternal 185 kb deletion. Many of which overlie regions of high regulatory potential (peaks along the “ESPERR Regulatory Potential” track).
