# Supplementary material for: Genetic variation in MKL2 and decreased downstream PCTAIRE1 expression in extreme, fatal primary human microcephaly
Source: Clin Genet. 2013 Jun 18;85(5):423–32. doi: 10.1111/cge.12197 (PMC3929543; doi:10.1111/cge.12197)
Supplement: Supplementary file 2 — Fig. S2. Relative brain cortical gene expression. Relative expression of 27 genes that included those known to be involved in the SRF:MKL2 pathway, those previously identified in cases of primary microcephaly, and CPPED1 located in the upstream chromosome 16 deletion and the housekeeping genes ACTB and PPIB to normalize expression was measured using the Quantigene Plex 2.0 Assay (Affymetrix). Thirteen individuals were surveyed: three normal brain anatomy fetal controls, three affected probands, and seven fetal specimens with pathology-diagnosed microcephaly. For each specimen, total RNA was extracted from an aggregate of six FFPE cerebral cortex cross sections affixed to glass slides according to the manufacturer’s protocol. The average cross-sectional area was 27.6 cm2 (range 9.0–49.5). Lysates of each sample were prepared by incubating each sample at 65°C for 6 h with 1 min of full speed vortexing every 60 min. Target-specific probes were designed and provided by Affymetrix. Target hybridization and signal amplification were performed according to manufacturer’s protocol. Signal measurements were made using a Luminex instrument (Austin, TX). Transcripts from each gene in all samples were measured in triplicate, and results expressed as the geometric mean normalized to an aggregate of ACTB and PPIB expression. Controls were six fetal brain tissue specimens without a pathology diagnosis of microcephaly. ‘Family’ is an average of gene expression from all three affected probands. ‘Other’ is an average of 33 other fetal cases with a pathology diagnosis of microcephaly. *p < 0.05; **p < 0.005. The red * is for comparison of ‘other’ microcephaly cases to normal controls. [file cge0085-0423-sd2.doc]

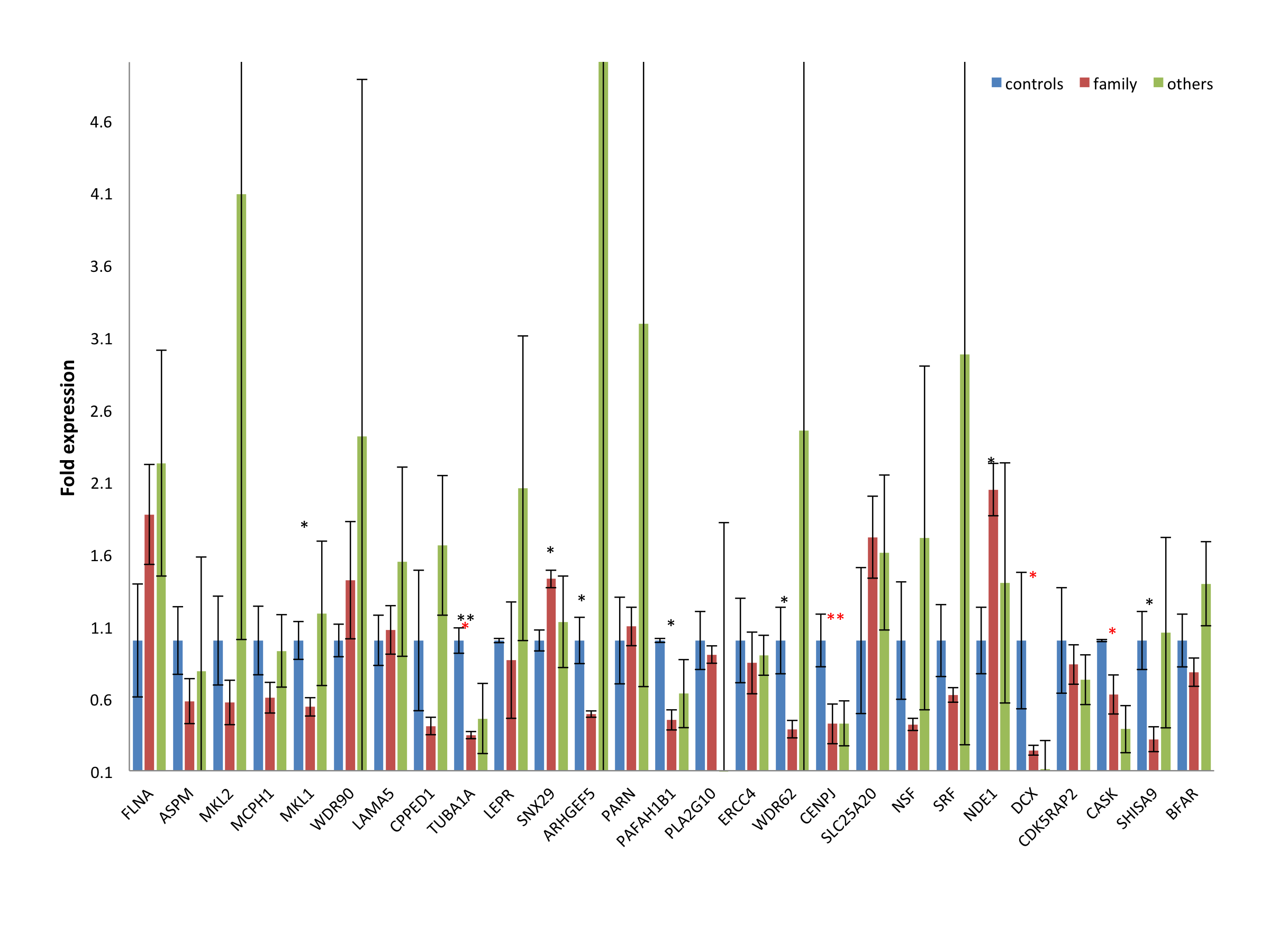


**Supplementary Figure 2. Relative brain cortical gene expression.** Relative expression of 27 genes that included those known to be involved in the *SRF:MKL2* pathway, those previously identified in cases of primary microcephaly, and *CPPED1* located in the upstream chromosome 16 deletion and the housekeeping genes *ACTB* and *PPIB* to normalize expression was measured using the Quantigene Plex 2.0 Assay (Affymetrix). Thirteen individuals were surveyed: three normal brain anatomy fetal controls, three affected probands, and seven fetal specimens with pathology-diagnosed microcephaly. For each specimen, total RNA was extracted from an aggregate of six FFPE cerebral cortex cross sections affixed to glass slides according to the manufacturer’s protocol. The average cross-sectional area was 27.6 cm^2^ (range 9.0 – 49.5). Lysates of each sample were prepared by incubating each sample at 65^o^C for six hours with one minute of full speed vortexing every 60 minutes. Target-specific probes were designed and provided by Affymetrix. Target hybridization and signal amplification were performed according to manufacturer’s protocol. Signal measurements were made using a Luminex instrument. Transcripts from each gene in all samples were measured in triplicate, and results expressed as the geometric mean normalized to an aggregate of *ACTB* and *PPIB* expression. Controls were six fetal brain tissue specimens without a pathology diagnosis of microcephaly. “Family” is an average of gene expression from all three affected probands. “Other” is an average of 33 other fetal cases with a pathology diagnosis of microcephaly. **p* < 0.05; ***p* < 0.005. The red * is for comparison of “other” microcephaly cases to normal controls.
