# Supplementary figures and images for: Genetic variation in MKL2 and decreased downstream PCTAIRE1 expression in extreme, fatal primary human microcephaly
Source: Clin Genet. 2013 Jun 18;85(5):423–32. doi: 10.1111/cge.12197 (PMC3929543; doi:10.1111/cge.12197)

**Supplementary Table 1.** Based positions analyzed in the paternal sperm exome.


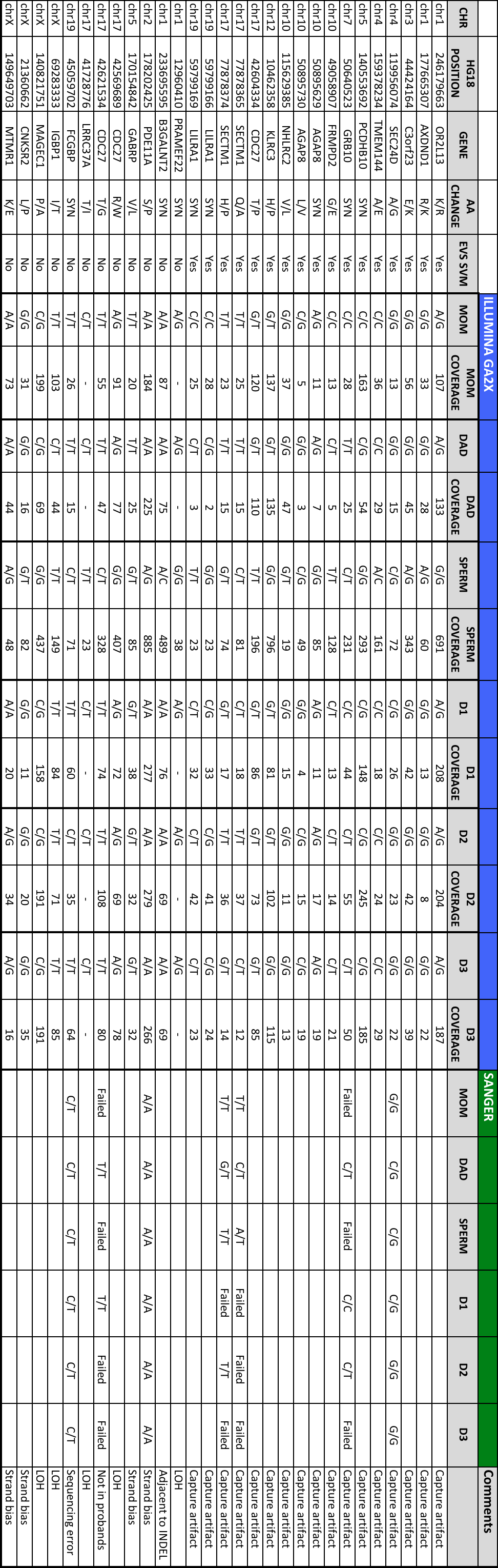

Supplement: Supplementary file 3 — Table S1. Based positions analyzed in the paternal sperm exome. [file cge0085-0423-sd3.doc]

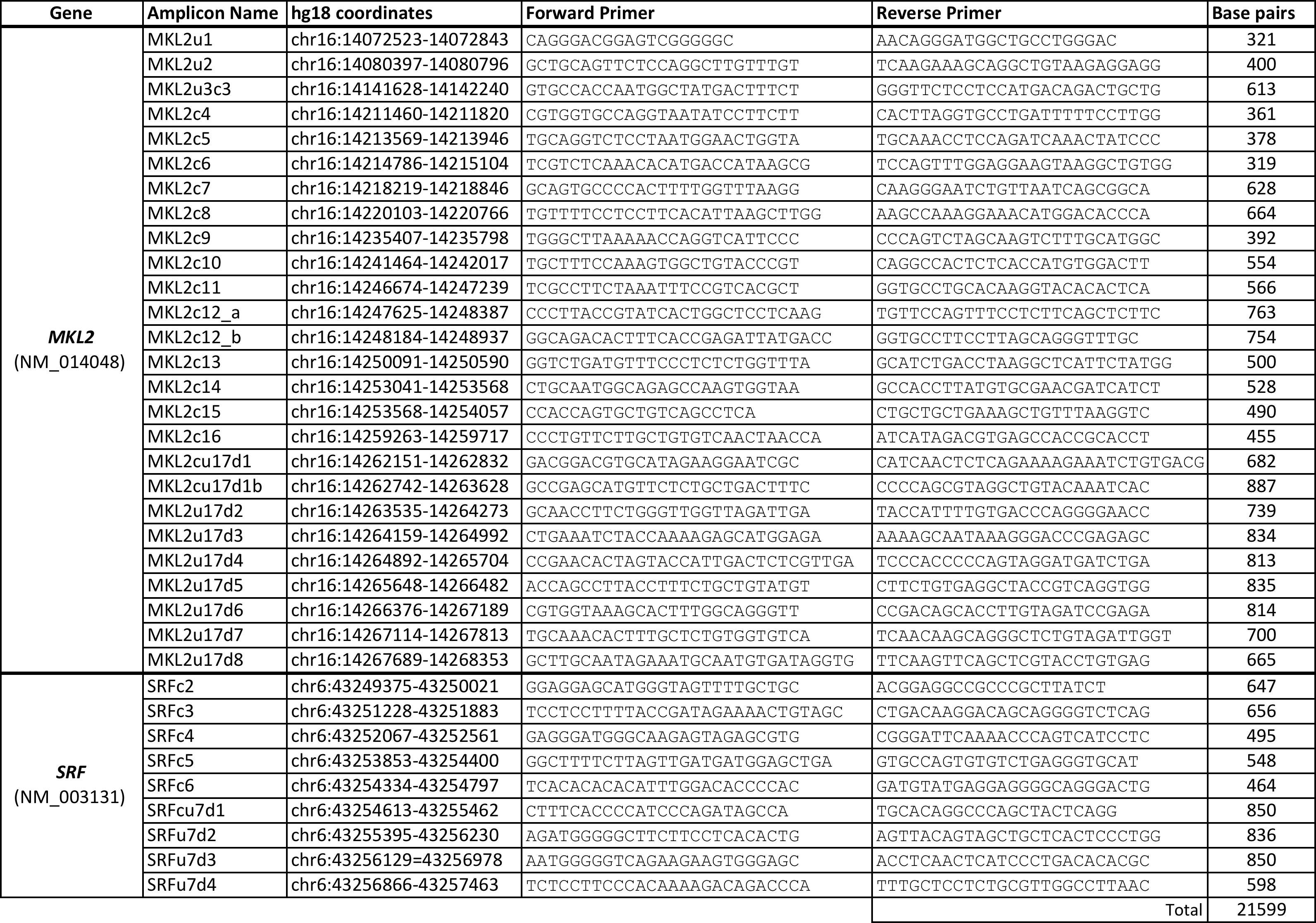


**Supplemental Table 3.** PCR primers for candidate region amplification and Sanger sequencing.

Supplement: Supplementary file 5 — Table S3. PCR primers for candidate region amplification and Sanger sequencing. [file cge0085-0423-sd5.doc]
