# Supplementary material for: Genetic variation in MKL2 and decreased downstream PCTAIRE1 expression in extreme, fatal primary human microcephaly
Source: Clin Genet. 2013 Jun 18;85(5):423–32. doi: 10.1111/cge.12197 (PMC3929543; doi:10.1111/cge.12197)
Supplement: Supplementary file 4 — Table S2. Sequencing results summary of MKL2 and SRF from 51 unrelated and geographically distinct primary microcephaly cases. Twelve cases were found to harbor sequence variants. The minor allele frequency, conservation (PhyloP) score and 7-species regulatory potential (ESPERR) score are listed. [file cge0085-0423-sd4.doc]

| Individual | Absolute position (hg18) | Reference Allele | Minor Allele | Genotype | Function | MAF (%) | PhyloP score | ESPERR score | Accession number |
| --- | --- | --- | --- | --- | --- | --- | --- | --- | --- |
| ***MKL2* (NM_014048) variants** (chromosome 16) | | | | | | | | | |
| 1 | 14248094 | C | T | HET | Synonymous* | n/a | -0.82 | 0.05 | Novel |
| 2 | 14141991 | C | T | HOM | 5’ UTR* | 0.3 | 2.25 | 0.27 | rs140275336 |
| 3 | 14080757 | C | A | HOM | Noncoding | 26.8 | -1.58 | 0.13 | rs13334806 |
|  | 14211676 | A | C | HET | Noncoding | 12.0 | -1.30 | 0.03 | rs226779 |
|  | 14213611 | T | A | HOM | Noncoding | 6.7 | 1.21 | 0 | rs13337739 |
|  | 14213620 | G | A | HET | Noncoding | 31.2 | 0.65 | 0 | rs226778 |
|  | 14218536 | T | C | HET | Synonymous | 10.1 | 2.85 | 0.25 | rs226788 |
|  | 14220534 | T | C | HET | Noncoding | 15.3 | -1.64 | 0 | rs226786 |
|  | 14253926 | G | C | HOM | Noncoding | 38.5 | -0.88 | 0 | rs30143 |
|  | 14264421 | A | C | HET | 3’ UTR | 15.0 | 0.34 | 0.07 | rs30129 |
| 4 | 14266706 | G | A | HET | 3’UTR | 31.9 | -0.96 | 0.12 | rs30123 |
| 5 | 14253402 | A | G | HET | Noncoding | 41.8 | 0.29 | 0.11 | rs30144 |
|  | 14253926 | G | C | HET | Noncoding | 38.5 | -0.88 | 0 | rs30143 |
|  | 14263822 | G | C | HET | 3’ UTR | 2.3 | 0.43 | 0.06 | rs76522200 |
|  | 14266706 | G | A | HET | 3’ UTR | 31.9 | -0.96 | 0.12 | rs30123 |
| 6 | 14218369 | C | T | HET | Noncoding | n/a | -1.04 | 0 | Novel |
| 7 | 14263822 | G | C | HET | 3’ UTR | 2.3 | 0.43 | 0.06 | rs76522200 |
| 8 | 14263874 | T | C | HET | 3’ UTR | 15.8 | -0.90 | 0.29 | rs30127 |
|  | 14264421 | A | C | HET | 3’ UTR | 15.0 | 0.34 | 0.07 | rs30129 |
|  | 14266734 | T | A | HET | 3’ UTR | 13.7 | -1.25 | 0.17 | rs30122 |
| 9 | 14141991 | C | T | HET | 5’ UTR* | 0.3 | 2.25 | 0.27 | rs140275336 |
| ***SRF* (NM_003131) variants** (chromosome 6) | | | | | | | | | |
| 10 | 43249872 | G | A | HET | Noncoding | n/a | -0.92 | 0.26 | Novel |
| 11 | 43251317 | G | T | HET | Noncoding | 2.9 | 1.15 | 0 | rs59063320 |
| 12 | 43256479 | C | T | HOM | 3’ UTR | n/a | 2.71 | 0.06 | Novel |

**Supplementary Table 2.** Sequencing results summary of *MKL2* and *SRF* from 51 unrelated and geographically distinct primary microcephaly cases. Twelve cases were found to harbor sequence variants. The minor allele frequency, conservation (PhyloP) score and 7-species regulatory potential (ESPERR) score are listed. Asterisks (*) denote abolishment of a methylated cytosine according to the UCSF brain methylation database displayed on the UCSC Genome Browser (http://genome.ucsc.edu/). MAF, minor allele frequency.
